# Effect of the Deletion of Genes Related to Amino Acid Metabolism on the Production of Higher Alcohols by *Saccharomyces cerevisiae*

**DOI:** 10.1155/2020/6802512

**Published:** 2020-11-05

**Authors:** Ya-Ping Wang, Xiao-Qing Wei, Xue-Wu Guo, Dong-Guang Xiao

**Affiliations:** ^1^Key Laboratory of Industrial Fermentation Microbiology (Tianjin University of Science and Technology), Ministry of Education, Tianjin 300457, China; ^2^Tianjin Industrial Microbiology Key Laboratory, College of Biotechnology, Tianjin University of Science and Technology, Tianjin 300457, China

## Abstract

The higher alcohols produced by *Saccharomyces cerevisiae* exert remarkable influence on the taste and flavour of Chinese Baijiu. In order to study the regulation mechanism of amino acid metabolism genes on higher alcohol production, eight recombinant strains with amino acid metabolism gene deletion were constructed. The growth, fermentation performance, higher alcohol production, and expression level of genes in recombinant and original *α*5 strains were determined. Results displayed that the total higher alcohol concentration in *α*5*ΔGDH1* strain decreased by 27.31% to 348.68 mg/L compared with that of *α*5. The total content of higher alcohols in *α*5*ΔCAN1* and *α*5*ΔGAT1* strains increased by 211.44% and 28.36% to 1493.96 and 615.73 mg/L, respectively, compared with that of *α*5. This study is the first to report that the *CAN1* and *GAT1* genes have great influence on the generation of higher alcohols. The results demonstrated that amino acid metabolism plays a substantial role in the metabolism of higher alcohols by *S. cerevisiae.* Interestingly, we also found that gene knockout downregulated the expression levels of the knocked out gene and other genes in the recombinant strain and thus affected the formation of higher alcohols by *S. cerevisiae*. This study provides worthy insights for comprehending the metabolic mechanism of higher alcohols in *S. cerevisiae* for Baijiu fermentation.

## 1. Introduction

**Figure 1:**
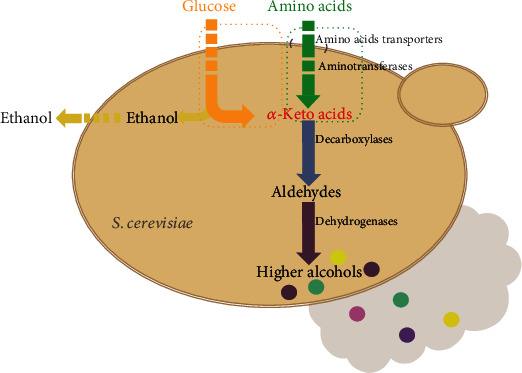
The pathway of higher alcohol formation in *S. cerevisiae*. Two pathways for yeast to metabolize higher alcohols are described in the figure. Amongst them, the orange dotted box represents the biosynaptic pathway, and the green dotted box represents the Ehrlich pathway. The colored dots in the figure represent n-propanol, isobutanol, isoamyl alcohol, phenylethanol, and other higher alcohols.

Higher alcohol is one of the important flavour substances in Baijiu [1] that are involved in the formation of Baijiu taste and flavour [2]. Baijiu has 12 flavour types in China. Each flavour type of Baijiu has its unique style because of its unique content of higher alcohols and other flavour substances. For example, Maotai jiu has higher content of higher alcohols, a prominent Maotai flavour and a heavy taste, whereas Fen jiu (traditional Baijiu with light aroma) has less higher alcohol content, is fresh and clean, and tastes light and pure [3]. The appropriate content of higher alcohols brings unique aroma and mellow taste to Chinese Baijiu [4]. However, excessive higher alcohol concentration will lead to fusel oil taste and is potentially harmful to human health as it may cause hangover and cerebral paralysis [5, 6]. According to reports, higher alcohols are generated by yeast during the fermentation of alcohol [7]. Higher alcohols are produced by the decarboxylation and dehydrogenation of *α*-ketoacids in yeast cells [8]. As displayed in Figure 1, the metabolic pathway for *Saccharomyces cerevisiae* to generate higher alcohols is divided into the catabolic pathway (Ehrlich pathway) and biosynthetic pathway according to the different sources of *α*-ketoacids [9, 10].

Amino acids as a nitrogen source utilised by yeast play a critical role in the formation of higher alcohols through the Ehrlich pathway [11], and their metabolism is divided into the following steps. Firstly, amino acids are transported from the fermentation substrate to yeast cells through amino acid transporters [12–14]. For example, branched amino acids can be transported by *BAP2*-encoded branched amino acid-permeable enzyme [15], and aromatic amino acids are absorbed by aromatic amino acid-permeable enzymes. Then amino acids generate corresponding *α*-ketoacids under the action of aminotransferases, such as the branched chain amino acid (BCAA) transaminases encoded by *BAT1* and *BAT2* genes [16–18] and the aromatic amino acid transaminases encoded by *ARO8* and *ARO9* genes [19]. Then, *α*-ketoacids generate corresponding aldehydes under the action of decarboxylases. The metabolism of amino acids involved in this pathway can cross each other because of the wide substrate specificity of decarboxylase. Aldehydes can catalyse the formation of higher alcohols under the action of various alcohol dehydrogenases [17]. Finally, higher alcohols are discharged into the fermentation broth through simple infiltration from yeast cells. Many studies have demonstrated that amino acids are absorbed by yeast as a nitrogen source in the process of alcohol fermentation to allow fermentation to proceed as expected [20]. Amino acids in fermentation media are important for the growth of fermentation microorganisms and the formation of flavour compounds [21].

Amino acids are important in the formation of flavour compounds by yeast. The deficiency of amino acids in fermentation medium will cause yeast to synthesise the amino acids it needs by using the ketoacids produced in sugar metabolism; thus, the production of ethanol, higher alcohols, esters, and other flavour substances will change when amino acids are lacking [22–24]. Aoki et al. mutated Lu's yeast with nitrosoguanidine and obtained a strain with substantially decreased utilisation of leucine and phenylalanine [25]. The isoamyl alcohol and 2-phenyl ethanol contents of the mutated strain decreased by 65.96% and 90.70%, respectively, compared with those of the parent strain. The inactivation of *BAT2* gene in haploid yeast strain can reduce the concentration of isoamyl alcohol and isobutanol [26]. Pirkov et al. confirmed that the mutants of *ARO8* and *ARO9* genes of the knockout haploid *S. cerevisiae* could not catalyse the transamination of aromatic amino acids [19]. We found that the utilisation of amino acids by yeast has an important impact on the formation of higher alcohols by yeast. Although many studies have reported on the influence of amino acid metabolism on the production of higher alcohols by *S. cerevisiae*, only a few have reported on the change of higher alcohol content in *S. cerevisiae* as a consequence of the interaction of genes involved in amino acid metabolism. Therefore, we focused on the effects and interaction of these genes on the higher alcohol production of yeast.

In our study, the effect of amino acid metabolism-related genes, namely, *AGP1* (encodes amino acid transporter), *GAD2* (encodes glutamic acid decarboxylase), *ARO80* (encodes amino acid transaminase transcriptional activator), *GAT1* (encodes the transcriptional regulatory protein of nitrogen breakdown repression mechanism), *CAN1* (encodes arginine permeability enzyme), *BAP2* (encodes BCAA permeability enzyme), *GDH1* (encodes glutamic acid dehydrogenase), and *BAT2* (encodes BCAA transferase), on the concentration of higher alcohols in yeast was investigated by individually deleting each gene. The gene expression level, growth, and fermentation performance, as well as the higher alcohols produced in fermentation of the parental and recombinant strains, were determined. The regulation of amino acid metabolism in yeast on the formation of flavour compounds was analysed to provide guidance in the construction of industrial *S. cerevisiae* strains with desired higher alcohol concentration for Chinese Baijiu fermentation.

## 2. Materials and Methods

### 2.1. Strains, Plasmids, and Primers

All the strains and plasmid used in this study are shown in Table 1.

### 2.2. Medium and Culture Conditions

Yeast strains were cultured in yeast extract-peptide-dextrose (YEPD) medium (2% peptone, 2% glucose, and 1% yeast extract) in a 30°C incubator. The YEPD medium (Promega, Madison, WI, USA) with 1 mg/mL G418 antibiotic (Promega, Madison, WI, USA) was used to screen the transformed recombinant strains using the *KanMX* gene. All solid culture media were added with 2% agar powder (Solarbio, Beijing, China).

Sorghum hydrolysate was used for the culture of primary and secondary seed cultures of yeast cells according to the method in Li et al.'s literature [27]. Firstly, sorghum powder was weighed, added with hot water (65°C) with a material-to-liquid ratio of 1 : 4, and stirred evenly for gelatinisation. Then, high-temperature-resistant *α*-amylase (the addition amount was 3 U/g, and enzyme activity was 2 × 10^4^ U/mL) was added. The mash was kept in an 85–90°C water bath for 1 h to liquefy, during which the mash was stirred from time to time. Then, the temperature of mash was reduced to 60°C, and saccharifying enzyme (the addition amount was 90 U/g, and enzyme activity was 10 × 10^4^ U/mL) was added. The mash was placed in a 55–60°C water bath for 20 h and stirred evenly. Then, saccharification was carried out. After saccharification, the temperature of the hydrolysate was reduced to 40°C, acid protease (the addition amount was 4 U/g, and enzyme activity was 2 × 10^4^ U/mL) was added, and the mixture was let stand for 30 min. Lastly, the hydrolysate was filtered using two layers of gauze and adjusted with water until the sorghum juice had a sugar content of 20°Bx. Each 145 mL of sorghum juice was dispensed into a 250 mL flask, then sterilised at 115°C for 20 min and cooled to room temperature. The second precultured yeast (5 mL) was inoculated to the prepared sorghum hydrolysate medium with an inoculum density of approximately 5 × 10^8^ cells/mL. All fermentations were performed in triplicate.

### 2.3. Construction of Recombinant Strain with Single Gene Deletion

Gene deletion in parental strain *α*5 was carried out by integrating the *KanMX* box, which was amplified by polymerase chain reaction (PCR) with target-K-U/target-K-D as primers and pUG6 plasmid as a template. The PCR primers are listed in Supplemental Table S1 and were designed on the basis of the *S. cerevisiae* S288c genome sequence in the National Centre for Biotechnology Information website (http://www.ncbi.nlm.nih.gov/). The upstream and downstream homologous fragments of the target gene were amplified by PCR using target-FA-U/target-FA-D and target-FB-U/target-FB-D as primers and *α*5 genome as a template, respectively.

The homologous recombination of the fragment and yeast genome was implemented using the LiAc/SS carrier DNA/PEG method [28]. YEPD solid medium mixed with 1 mg/mL G418 was used to preliminary screen the transformants. The exact integration of the *KanMX* box in a single colony was verified by diagnostic oligonucleotide PCR with verification primers (target-1-U/-1-D and target-2-U/target-2-D). Then, the right single colony was selected for purification, and second validation was carried out. Finally, the correct single colony is the right recombinant strain we constructed.

### 2.4. RNA Extraction and Quantitative Real-Time PCR (RT-qPCR)

Total RNA was extracted by a yeast RNA isolation kit and then reverse-transcribed using a PrimeScript™ RT reagent kit. Changes in gene expression level were assessed by RT-qPCR with SYBR® Premix Ex Taq™ II test kit (Tli RNaseH Plus). The kits were purchased from Takara (Takara Biotechnology, Dalian, China). The PCR primers (target-U/target-D) listed in Supplementary Table S1 were synthesised by GENEWIZ (Suzhou, China). The PCR procedure was set according to that reported by Li and Chen [1]. The result was analysed quantitatively using 2^−*ΔΔ*Ct^ method, and the actin gene (*UBC6*) was used as the housekeeping gene.

### 2.5. Growth Curve Determination

Yeast strains were inoculated into 5 mL of YEPD liquid culture medium and cultured in a 30°C shaking bed incubator for 12 h. Then, 40 *μ*L of yeast cell fluid was added into 360 *μ*L of YEPD liquid medium, and the mixture was transferred to a 96-well plate. Optical density at 600 nm was measured every 30 min by a Bioscreen automated growth curve analysis system (OY Growth Curves Ab Ltd., Helsinki, Finland).

### 2.6. Fermentation Analysis

Fermentation experiment was carried out in a 250 mL flask with 150 mL of the separated sorghum hydrolysate. The second precultured yeast seed liquid was inoculated to the sorghum hydrolysate medium. The inoculum density was approximately 5 × 10^8^ cells/mL. Fermentation was carried out in a 30°C incubator, and the hydrolysate was weighed every 12 h. Fermentation was concluded when the weight loss was less than 0.1 g. The hydrolysate was distilled, and the content of higher alcohols and other flavour substances in the distillate was determined by gas chromatography method according to Ma et al.'s report [29].

### 2.7. Statistical Data Analysis

All experiments were repeated three times. The growth curves and other charts were drawn using the Origin 2018 software. The heat map was drawn using Excel 2016 and Adobe Illustrator CS4 software. Data calculation and processing were performed in Excel 2016, and the results are displayed as the mean ± standard deviation. The significance of the difference between experimental and control groups was analysed using a *t*-test (^★★^*P* < 0.01, ^★^*P* < 0.05).

## 3. Results

### 3.1. Effect of Amino Acid Metabolism-Related Gene Deletion on the Growth and Fermentation Performance of *S. cerevisiae*

**Figure 2:**
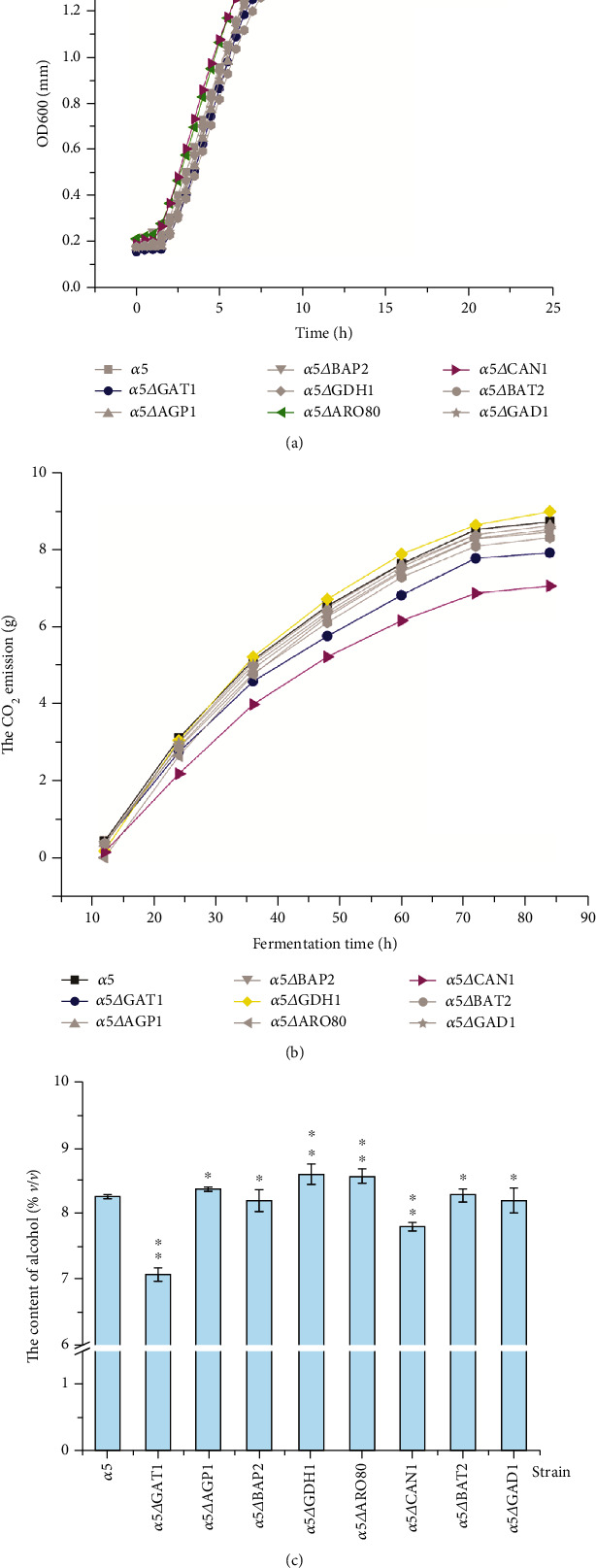
Growth and fermentation properties of recombinant strains and *α*5. (a) Growth curve of recombinant strains and *α*5. (b) The CO_2_ emission of recombinant strains and *α*5. (c) The alcohol content of recombinant strains and *α*5. The error bar represents the standard deviation of three repeated fermentation experiment data. The significant difference between the recombinant strain and the parent strain was tested and confirmed by Student's *t*-test (^★★^*P* < 0.01, ^★^*P* < 0.05).

The growth and fermentation performance of *S. cerevisiae* were monitored during gene knockout. The growth curve, fermentation curve, and alcohol production are shown in Figures 2(a)–2(c), respectively. The growth rate of each recombinant strain showed a slight difference compared with that of the parent strain *α*5 (Figure 2(a)). The growth curve with larger change range compared with *α*5 was labelled with colour. The results in Figure 2(a) demonstrate that *α*5*ΔCAN1* mutant entered the stable stage earlier, the final biomass of *α*5*ΔARO80* was higher, and the final biomass of *α*5*ΔGAT1* was lower, compared with those of *α*5. The growth curves of the other mutants were similar to that of *α*5. The results showed that the inactivation of these genes exerts no noticeable effect on the growth performance of *S. cerevisiae.* The loss of CO_2_ in the fermentation medium was weighed every 12 h during the whole fermentation process to assess the fermentation properties of the recombinant strains (Figure 2(b)). As described in Figure 2(b), the CO_2_ emission of the *α*5*ΔCAN1* strain was the lowest amongst the strains, and the *α*5*ΔGAT1* strain had lower CO_2_ emission compared with the parent strain *α*5. The CO_2_ emissions of the *α*5*ΔCAN1* and *α*5*ΔGAT1* strains decreased by 19.05% (i.e., 7.07 g) and 9.13% (i.e., 7.93 g) compared with that of the parent strain *α*5, respectively. The recombinant strain *α*5*ΔGDH1* had the highest CO_2_ emission of 9.00 g, which was 3.09% higher than that of *α*5. In addition, the CO_2_ emission in most of the recombinant strains had no obvious difference with that of the parent strain *α*5. In the fermentation process, the weight loss of CO_2_ was reflected in the formation of alcohol. Significant differences were seen in the alcohol content amongst the recombinant strains and *α*5 (Figure 2(c)). As shown in Figure 2(c), the alcohol contents of *α*5*ΔGAT1* and *α*5*Δ CAN1* remarkably decreased by 14.34% (i.e., 7.07% (*v*/*v*)) and 5.45% (i.e., 7.80% (*v*/*v*)) compared with that of the original strain *α*5, respectively. The alcohol content of strain *α*5*ΔGDH1* increased by 4.24% to 8.60% (*v*/*v*) compared with that of *α*5, and this result was consistent with the result of CO_2_ emission in Figure 2(b). The findings in Figure 2(c) indicate that *GAT1*, *CAN1*, and *GDH1* deletions have different influences on the growth and fermentation performance of *S. cerevisiae α*5. *α*5*ΔGAT1*, *α*5*ΔCAN1*, and *α*5*ΔGDH1* strains showed significant difference in alcohol content compared with that of *α*5. We consider that the reason for the difference in the alcohol consumption of the recombinant strains is the deletion of the gene related to amino acid transportation, which led to the diversity in the utilisation of amino acids by yeast and finally reflected in the discrepancy of alcohol yield.

### 3.2. Effect of Amino Acid Metabolism-Related Gene Deletion on the Gene Expression Level in *S. cerevisiae*

**Figure 3:**
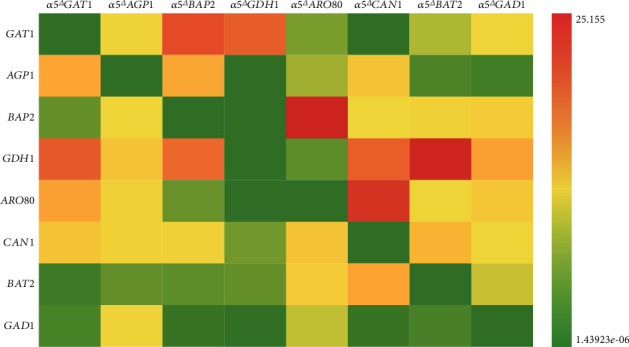
Determination of genes expression levels in recombinant strains and *α*5. The gene expression level in each recombinant strain was measured, compared with *α*5. With the change fold as the data, the heat map of the expression level of each gene in the recombinant strain was drawn by Excel 2016. Each row represents the expression level of each target gene in each recombinant strain, compared with the parent strain *α*5. Red indicates that the expression level of the target gene is upregulated, while green indicates that it is downregulated, respectively, compared with that of *α*5. The deeper the color, the larger the up/downregulation. Amongst them, the maximum upregulation multiple was 25.155, and the maximum downregulation multiple was 1.43923*e*^−06^, which was compared with *α*5.

The gene expression levels of the recombinant strains and the parent strain *α*5 were measured by RT-qPCR to explore the interaction amongst the genes (Figure 3). Diagonally, from the upper left corner to the lower right corner of Figure 3, we can see that gene knockout reduced the expression level of the knocked out gene in *S. cerevisiae*. Interestingly, amino acid metabolism-related genes had various expression levels in these recombinant strains. For example, the *GDH1* gene was highly expressed in *α*5*ΔGAT1* and *α*5*ΔCAN1* strains. The expression of *CAN1* and *BAP2* genes was upregulated in *α*5△*AGP1* strain compared with those in *α*5. Moreover, the expression of *CAN1* and *BAP2* was downregulated in strain *α*5*ΔGDH1* compared with those in *α*5. *BAT2* gene was highly expressed in the recombinant strain *α*5*ΔCAN1* compared with that in *α*5. The discrepancy in the gene expression levels of different recombinant strains may have certain effects on their metabolism. Gene interaction changes the growth and fermentation properties of the recombinant strains and thus regulates the higher alcohol concentration in yeast.

### 3.3. Effect of Amino Acid Metabolism-Related Gene Deletion on the Formation of Higher Alcohols of Recombinant Strains

**Figure 4:**
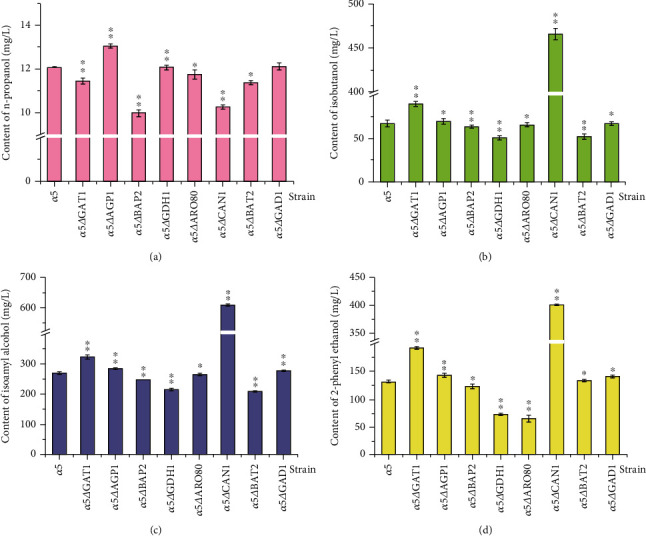
The formation of higher alcohol of recombinant strains and *α*5. (a) The n-propanol content in recombinant strains and the parental strain *α*5. (b) The isobutanol content of recombinant strains and *α*5. (c) The isoamyl alcohol content of recombinant strains and *α*5. (d) The 2-phenyl ethanol content of recombinant strains and *α*5. The error bar represents the standard deviation of three repeated fermentation experiment data. The significant difference between the recombinant strain and the parent strain was verified by Student's *t*-test (^★★^*P* < 0.01, ^★^*P* < 0.05).

The concentrations of n-propanol, isobutanol, isoamyl alcohol, and 2-phenyl ethanol in recombinant strains and *α*5 were detected to study the influence of the knockout of amino acid metabolism-related genes on the production of higher alcohols. Remarkable differences were noticed in the higher alcohol concentrations amongst the recombinant strains and *α*5 (Figures 4(a)–4(d)). As shown in Figure 4(a), the concentration of n-propanol in strain *α*5*ΔAGP1* increased by 8.02% to 13.06 mg/L compared with that of the parent strain *α*5. The n-propanol contents of the other recombinant strains presented decreased by varying degrees. Amongst them, the n-propanol content of strain *α*5*ΔBAP2* decreased by 17.37% (i.e., 9.99 mg/L) compared with that of the parent strain *α*5. The production of n-propanol by *AGP1* deletion haploid strain *α*5*ΔAGP1* and *BAP2* deletion haploid strain *α*5*ΔBAP2* had significant differences. The isobutanol content of *α*5*ΔCAN1* strain was the highest amongst the strains and reached 465.98 mg/L, which was higher by 596.64% compared with that of *α*5 (Figure 4(b)). In addition, as shown in Figure 4(b), the isobutanol contents of strains *α*5*ΔGDH1* and *α*5*ΔBAT2* decreased by 24.67% and 22.57% to 50.39 and 51.79 mg/L, respectively, compared with that of *α*5. Significant differences were found in the production of isobutanol by *α*5*ΔAGP1* and *α*5*ΔBAP2* compared with that of *α*5. The isoamyl alcohol content of *BAT2* gene mutant decreased (Figure 4(c)), and this result was consistent with previously reported results [30]. The isoamyl alcohol contents of strains *α*5*ΔGAT1* and *α*5*ΔCAN1* increased by 19.75% (i.e., 322.91 mg/L) and 126.10% (i.e., 609.71 mg/L), respectively, compared with that of *α*5. There are significant differences in the production of isoamyl alcohol by *α*5△*BAT2*, *α*5△*GAT1*, and *α*5△*CAN1*, compared with that of *α*5. From Figures 4(b) and 4(c), we found that the amount of isobutanol and isoamyl alcohol in the single gene knockout recombinant strain had the same trend. It is noteworthy that the 2-phenyl ethanol content by the strain *α*5△*ARO80* and *α*5△*GDH1* decreased significantly by 50.72% (i.e., 64.59 mg/L) and 44.82% (i.e., 72.32 mg/L), respectively, compared with that of *α*5 (Figure 4(d)). Figure 4(d) also shows that the 2-phenyl ethanol content of recombinant strain *α*5*ΔGAT1* increased by 46.16% to 191.56 mg/L compared with that of *α*5. Furthermore, similar to isobutanol and isoamyl alcohol yields, the 2-phenyl ethanol content of *α*5*ΔCAN1* strain increased by 205.91% to 400.93 mg/L. The 2-phenyl ethanol production of *α*5*ΔGAT1* and *α*5*ΔCAN1* was significant different compared with that of *α*5.

### 3.4. Effect of Amino Acid Metabolism-Related Gene Deletion on the Formation of Total Higher Alcohols of Recombinant Strains

**Figure 5:**
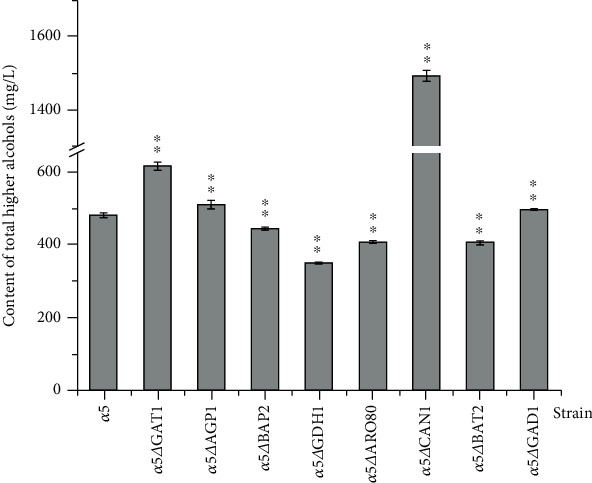
Content of total higher alcohols in the recombinant strains and *α*5. The significant difference between the recombinant strain and the parent strain was verified by Student's *t*-test (^★★^*P* < 0.01, ^★^*P* < 0.05).

The impact of amino acid metabolism-related gene deletion on total higher alcohol concentration in eight recombinant strains was further analysed. Significant differences were noticed in the total higher alcohol contents amongst the recombinant strains and *α*5 (Figure 5). According to the content of total higher alcohols (Figure 5), isobutanol, isoamyl alcohol, and 2-phenyl ethanol are the main components of higher alcohols; therefore, the change in total higher alcohol content is consistent with the changes in the three higher alcohols. The total higher alcohol content of the *α*5△*GDH1* strain decreased by 27.31% to 348.68 mg/L compared with that of *α*5. The total higher alcohol contents of *α*5*ΔCAN1* and *α*5*ΔGAT1* strains increased by 211.44% (i.e., 1493.96 mg/L) and 28.36% (i.e., 615.73 mg/L), respectively, compared with that of *α*5. Besides, the total higher alcohol contents of the other recombinant strains had little change.

## 4. Discussion

Higher alcohols produced by yeast have an important effect on the flavour and taste of alcoholic beverages [31, 32]. In the Ehrlich pathway, the utilisation of amino acids by yeast plays a critical role in the formation of higher alcohols [33]. In this research, we concentrated on the effect of the inactivation of genes related to amino acid metabolism on the growth, fermentation performance, and higher alcohol formation of *S. cerevisiae*. Eight mutants with single gene (*GAT1*, *AGP1*, *BAP2*, *GDH1*, *ARO80*, *CAN1*, *BAT2*, and *GAD1*) deletion were constructed. The gene expression level, growth properties, fermentation performance, and higher alcohol formation in each recombinant strain were determined. We reported for the first time that the inactivation of the *GDH1* gene would reduce the total higher alcohol content in yeast and the knockout of *CAN1* and *GAT1* genes would increase the formation of total higher alcohols in *S. cerevisiae*. Moreover, we confirmed the influence of gene interaction on the yield of higher alcohols in *S. cerevisiae*.

The alcohol concentration of the *α*5*ΔGDH1* strain increased by 4.24% compared with that of *α*5 (Figure 2(c)). *S. cerevisiae* can use the nutrients in the culture medium for alcohol fermentation. The utilisation of nutrients directly affects the speed and yield of alcohol production by yeast [34, 35]. A study demonstrated that the inactivation of the *GDH1* gene can eliminate the dependence of the glutamate metabolic pathway on NADPH and make yeast specifically dependent on the metabolism of NADH to change the metabolic flow of NADH in yeast cells, reduce the formation of glycerol in the fermentation process of *S. cerevisiae*, increase the conversion of carbon flow to ethanol, and improve the production of ethanol in the fermentation process by yeast [36]. Besides, according to the changes in the gene expression levels of amino acid metabolism-related genes in each recombinant strain, we believe that the formation of higher alcohols in *S. cerevisiae* is not the result of a single gene but the interaction between genes that regulate the formation of higher alcohols. For example, the knockout of the *GDH1* gene will lead to the increase of alcohol content in yeast (Figure 2(c)); thus, the expression of the *GDH1* gene is negatively related to alcohol production in yeast. We noticed that the *GDH1* expression levels in *α*5*ΔGAT1* and *α*5*ΔCAN1* strains were substantially upregulated than that in *α*5 and the alcohol contents of *α*5*ΔGAT1* and *α*5*ΔCAN1* strains remarkably decreased (Figure 2(c)). Therefore, the expression of the *GDH1* gene in *α*5*ΔGAT1* and *α*5*ΔCAN1* strains regulates alcohol production. We can explain the changes in fermentation performance caused by gene deletion by combining the gene expression levels in recombinant strains with the results of fermentation performance. The deletion of the *GDH1* gene would reduce the formation of total higher alcohols by *S. cerevisiae*. Our result showed that the total higher alcohol content in *α*5*ΔGDH1* strain decreased by 27.31% to 348.68 mg/L compared with that of *α*5 (Figure 5). *GDH1* encodes glutamate dehydrogenase, and its transcription and expression are regulated by carbon and nitrogen sources. Leu3p and Gcn4p are the two key regulatory proteins that participate in the transcription regulation of BCAA metabolism in yeast. Leu3p is a pathway-specific regulator that regulates six genes involved in BCAA metabolism, including *GDH1* [37]. We speculate that the knockout of *GDH1* affects the metabolism of BCAA in yeast and leads to the change in higher alcohol production by yeast.

Figure 5 shows that the total higher alcohol content of *α*5*ΔCAN1* increased by 211.44% to 1493.96 mg/L compared with that of *α*5. Notably, the knockout of *CAN1* reduced the n-propanol content of the recombinant strain by 15.14% compared with that of the parent strain *α*5 (Figure 4(a)). By contrast, isobutanol, isoamyl alcohol, and 2-phenyl ethanol increased by 596.64%, 126.10%, and 205.91%, respectively, in *α*5*ΔCAN1* compared with *α*5 (Figure 4(b)). The effect of *CAN1* deletion on higher alcohol production by yeast is rarely reported. *CAN1* encodes arginine transaminase, which regulates the utilisation of arginine by *S. cerevisiae* [38]. We suspect that the absence of *CAN1* leads to the decrease in the utilisation rate of arginine in yeast, which affects the nitrogen metabolism of yeast and ultimately affects the fermentation performance and flavour quality of the final product. In addition, we noticed that the expression level of *BAT2* was upregulated in the recombinant strain *α*5*ΔCAN1* compared with *α*5 (Figure 3), and the isobutanol and isoamyl alcohol contents in *α*5*ΔCAN1* strain were also higher than those of *α*5 (Figures 4(b)–4(c)). Therefore, according to the results of gene expression level and higher alcohol concentration in the recombinant strain, we speculate that the change in flavour substances in *α*5△*CAN1* is caused by the knockout of *CAN1* and the interaction of multiple genes, such as *BAT2*, which leads to the change in yeast fermentation performance. Interestingly, we also found that the n-propanol contents of strains *α*5*ΔCAN1* and *α*5*ΔBAP2* decreased substantially compared with that in *α*5 (Figure 4(a)). Hence, the downregulation of the expression of these two genes will reduce the production of n-propanol in yeast. We noticed that the n-propanol content in strain *α*5*ΔAGP1* increased markedly compared with that in *α*5 (Figure 4(a)), and the expression level of *CAN1* and *BAP2* in *α*5*ΔAGP1* was upregulated compared with those in *α*5 (Figure 3). We inferred that the rise in *CAN1* and *BAP2* expression led to the increase in n-propanol content of *α*5*ΔAGP1*. However, in *α*5*ΔGDH1*, the expression levels of *CAN1* and *BAP2* were downregulated than that of *α*5 (Figure 3), and the n-propanol content of *α*5*ΔGDH1* was slightly higher than that of its parent strain *α*5 (Figure 4(a)), which was in contrast to the result of strain *α*5*ΔAGP1*. We consider that the expression of genes in the recombinant strain cannot explain the change in the fermentation performance of all yeast.

The total higher alcohol content of *α*5△*GAT1* increased by 28.36% to 615.73 mg/L compared with that of *α*5. The effect of *GAT1* deletion on the generation of higher alcohols in yeast has not been reported. *GAT1* gene is the key regulator of nitrogen catabolism inhibition gene transcription in *S. cerevisiae* [39]. *GAT1* was isolated in the cytoplasm when a good nitrogen source is added in the fermentation medium. By contrast, *GAT1* is transferred to the nucleus when the poor nitrogen source is exhausted; this transfer leads to the activation of sensitive genes related to the inhibition of nitrogen catabolism [40]. This inhibition can inhibit the absorption of arginine and alanine by *S. cerevisiae* and stimulate the utilisation of BCAA and aromatic amino acids [40, 41]. We consider that *GAT1* deletion leads to the failure of the activation of nitrogen metabolism, which affects the absorption and utilisation of nitrogen sources by yeast in the fermentation process and thus affects the generation of higher alcohols. Notably, the 2-phenyl ethanol content of *α*5*ΔGAT1* increased by 46.16% to 191.56 mg/L compared with that of *α*5. We think that the consumption of aromatic amino acids by yeast leads to the increase in 2-phenyl ethanol synthesis. We also observed that the isobutanol and isoamyl alcohol contents of *α*5*ΔBAT2* decreased by 22.57% and 22.29% compared with that of *α*5, respectively (Figures 4(b) and 4(c)). The BCAA aminotransferase encoded by *BAT2* gene in yeast cytoplasm is responsible for catalysing the production of corresponding *α*-ketoacids from BCAA [12]. Li et al. took the recombinant strain *α*5-IAH1 as the parental strain and constructed a recombinant strain with *BAT2* gene deletion and *ATF1* gene overexpression simultaneously; the isobutanol and isoamyl alcohol content of this strain decreased by 59.24% and 70.89%, respectively, compared with that of the parent strain [27]. Styger et al. found that the inactivation of *BAT2* makes a big difference on the formation of isobutanol and isoamyl alcohol in *S. cerevisiae* [42]. Their finding is consistent with our conclusion that the knockout of *BAT2* gene can cut down the utilisation of BCAA in yeast and thus decrease the concentration of higher alcohols produced by yeast.

The 2-phenyl ethanol content of strain *α*5*ΔARO80* decreased substantially by 50.72% to 64.59 mg/L compared with that of *α*5. *Aro80*p, a transcription factor encoded by *ARO80*, responds to aromatic amino acids and activates *ARO9* and *ARO10* expression [43]. The absence of *ARO80* will prevent the expression of *ARO9* and *ARO10* from being activated. Kim et al. overexpressed the transcription factor *Aro80*p in *S. cerevisiae*, which remarkably upregulated the expression levels of *ARO9* and *ARO10*, and increased 2-phenyl ethanol production by 58% compared with those of the original strain [44]. The expression of *ARO80* is positively correlated with the assimilation of aromatic amino acids. The upregulated expression level of *ARO80* and the high 2-phenyl ethanol contents of *α*5*ΔGAT1* and *α*5*ΔCAN1* (Figures 3 and 4(d)) infer that the expression level of *ARO80* in *α*5*ΔGAT1* and *α*5*ΔCAN1* increased and led to the increase in the 2-phenylethanol contents of *α*5*ΔGAT1* and *α*5*ΔCAN1*. We speculate that single-gene deletion destroys the metabolism of one or several amino acids, but yeast may increase gene expression in other alternative pathways to make up for the decrease in nutrition utilisation caused by gene inactivation. The recombinant yeast strains with gene knockout and blocked expression may strengthen other metabolic pathways to compensate for the effect of gene deletion. The formation of flavour substances in *S. cerevisiae* is the result of the interaction of multiple genes, which is interesting and complex, and deserves further study. The interaction between genes helps us to understand the regulatory mechanism between amino acid transport and higher alcohol synthesis in *S. cerevisiae* and is also of great importance to guide in the engineering of bacteria with industrial application value.

## 5. Conclusion

Higher alcohol is one of the by-products produced by yeast in alcohol fermentation, and its content has an important impact on the flavour and taste of alcoholic beverages. The utilisation of amino acids by yeast plays an important role in the formation of higher alcohols. We studied the effects of amino acid metabolism-related gene deletion on yeast growth, fermentation performance, and higher alcohol formation. The interaction between amino acid metabolism-related genes was found and confirmed that gene interaction plays an important role in the regulation of higher alcohols. In particular, we found that the deletion of *CAN1* and *GDH1* has an important effect on the higher alcohol content of *S. cerevisiae*. Our study provides valuable insights into the mechanism of higher alcohol production by *S. cerevisiae* in liquor fermentation. This study provides a new idea for the breeding of *S. cerevisiae* suitable for liquor fermentation.

## Figures and Tables

**Table 1:** Strains and plasmid used in this study.

Strains or plasmids	Genotypic or construct	References or origin
AY15 CICC32315	Commercial Baijiu yeast strain	This lab
*α*5	MAT*α*, haploid yeast strain from AY15	This lab
Transformants		
*△GAT1*	*α*5*△GAT1::loxP-KanMX-loxP*	This study
*△AGP1*	*α*5*△AGP1::loxP-KanMX-loxP*	This study
*△BAP2*	*α*5*△BAP2::loxP-KanMX-loxP*	This study
*△GDH1*	*α*5*△GDH1::loxP-KanMX-loxP*	This study
*△ARO80*	*α*5*△ARO80::loxP-KanMX-loxP*	This study
*△CAN1*	*α*5△*CAN1::loxP-KanMX-loxP*	This study
*△BAT2*	*α*5*△BAT2::loxP-KanMX-loxP*	This study
*△GAD1*	*α*5*△gad1::loxP-KanMX-loxP*	This study
Plasmid		
pUG6	Kanr, containing *loxP-KanMX-loxP* disruption cassette	Güldener et al., 2002

## Data Availability

The data used to support the findings of the current study are available from the corresponding author on reasonable request.

## Supplementary Materials

Supplementary Materials Supplementary Table S1: primers used in the present study.
