# Supplementary material for: Effect of the Deletion of Genes Related to Amino Acid Metabolism on the Production of Higher Alcohols by Saccharomyces cerevisiae
Source: Biomed Res Int. 2020 Nov 5;2020:6802512. doi: 10.1155/2020/6802512 (PMC7665916; doi:10.1155/2020/6802512)
Supplement: Supplementary Materials — Supplementary Table S1: primers used in the present study. [file 6802512.f1.pdf]

Supplementary table S1 Primers used in the present study

Name	5′–3′ DNA sequence
**Oligos used for construction of gene disruption cassettes**	
GAP1-FA-U	TAACGATAACCGATGGACC
GAP1-FA-D	CCTGCAGCGTACGAAGCTTCAGCTGGACACCGTAATACTGATGGA
GAP1-FB-U	TATCAGATCCACTAGTGGCCTATGCAGCAACCGAGACCTGAAC
GAP1-FB-D	CACCCAACTGAAACTTATCC
GAP1-K-U	ACAGCACTATGAGTCGCACCAGCTGAAGCTTCGTACGCTGCAGG
GAP1-K-D	GTTCAGGTCTCGGTTGCTGCATAGGCCACTAGTGGATCTGATA
AGP1-FA-U	TCATCCAAACCGACACCT
AGP1-FA-D	CCTGCAGCGTACGAAGCTTCAGCTGACCTCCAAGCCAAAGAAA
AGP1-FB-U	TATCAGATCCACTAGTGGCCTATGCGCATCTTAGCCGTCTTATC
AGP1-FB-D	GAATCGTCGTTACTGTGGT
AGP1-K-U	TTTCTTTGGCTTGGAGGTCAGCTGAAGCTTCGTACGCTGCAGG
AGP1-K-D	GATAAGACGGCTAAGATGCGCATAGGCCACTAGTGGATCTGATA
BAP2-FA-U	AGGTAGTTGGAGCGACAG
BAP2-FA-D	CCTGCAGCGTACGAAGCTTCAGCTGTGGACACTTCCCATTAGAG
BAP2-FB-U	TATCAGATCCACTAGTGGCCTATGCATTAGTAGGTGCAAGGTGG
BAP2-FB-D	CAAAGGTGTATGTACGGTTT
BAP2-K-U	CTCTAATGGGAAGTGTCCACAGCTGAAGCTTCGTACGCTGCAGG
BAP2-K-D	CCACCTTGCACCTACTAATGCATAGGCCACTAGTGGATCTGATA
GDH1-FA-U	GTGTTTGTGGGCAGATTAT
GDH1-FA-D	CCTGCAGCGTACGAAGCTTCAGCTGCACCAGGCTTATTGATGAC
GDH1-FB-U	TATCAGATCCACTAGTGGCCTATGCGGTAAACGCATTTGTAACTC
GDH1-FB-D	ATGACCAGAAAGCCCACT
GDH1-K-U	GTCATCAATAAGCCTGGTGCAGCTGAAGCTTCGTACGCTGCAGG
GDH1-K-D	GAGTTACAAATGCGTTTACCGCATAGGCCACTAGTGGATCTGATA
ADO80-FA-U	GAGCAGCACAACTACCCT
ADO80-FA-D	CCTGCAGCGTACGAAGCTTCAGCTGTCCCACGACTAACTTGATA
ADO80-FB-U	TATCAGATCCACTAGTGGCCTATGCACCAAGACAACCGCACTA
ADO80-FB-D	GACAAAGAAAGCCCAGAA
ADO80-K-U	TATCAAGTTAGTCGTGGGACAGCTGAAGCTTCGTACGCTGCAGG
ADO80-K-D	TAGTGCGGTTGTCTTGGTGCATAGGCCACTAGTGGATCTGATA
CAN1-FA-U	GGCACCAAGAATAGAGTTT
CAN1-FA-D	CCTGCAGCGTACGAAGCTTCAGCTGAGCAGACGGAGTAGAAGC
CAN1-FB-U	TATCAGATCCACTAGTGGCCTATGCATCACTTGTTGCCTGTATC
CAN1-FB-D	GTCACTGTATCTGCTGCTT
CAN1-K-U	GCTTCTACTCCGTCTGCTCAGCTGAAGCTTCGTACGCTGCAGG
CAN1-K-D	GATACAGGCAACAAGTGATGCATAGGCCACTAGTGGATCTGATA
BAT2-FA-U	CTCATCTACTTCAAGAGACTGAG

BAT2-FA-D	CCTGCAGCGTACGAAGCTTCAGCTGATCGTTCTTAAAACTCGTGGAG
BAT2-FB-U	TATCAGATCCACTAGTGGCCTATGCAGTATCGCTATTGCTACGTAAAG
BAT2-FB-D	GCCTAAAGCCATCTGATAGGTAC
BAT2-K-U	CTCCACGAGTTTTAAGAACGATCAGCTGAAGCTTCGTACGCTGCAGG
BAT2-K-D	CTTTACGTAGCAATAGCGATACTGCATAGGCCACTAGTGGATCTGATA
GAD1-FA-U	CACCTATTTCCCGTGCTT
GAD1-FA-D	CCTGCAGCGTACGAAGCTTCAGCTGAATGTCAGACCCTACTATCAAC
GAD1-FB-U	TATCAGATCCACTAGTGGCCTATGCGCCTGGACAGTGATAGAAA
GAD1-FB-D	CAACGGGATAGGAATAAAA
GAD1-K-U	GTTGATAGTAGGGTCTGACATTCAGCTGAAGCTTCGTACGCTGCAGG
GAD1-K-D	TTTCTATCACTGTCCAGGCGCATAGGCCACTAGTGGATCTGATA

Oligos used for diagnostic purposes

GAT1-1-U	TTGACATTCTTCTGGCTTCC
GAT1-1-D	CCTCAGTGGCAAATCCTAA
GAP1-2-U	AATAACGGTTTGGTTGA
GAP1-2-D	TTGGCTGAATACTACTCCTT
AGP1-1-U	CTAACGAGCGAGCACA
AGP1-1-D	CAGTATAGCGACCAGCA
AGP1-2-U	GCAGACCGATACCAGG
AGP1-2-D	TTACACCGCAAGACCA
BAP2-1-U	ACATTTCTTACGGATTGG
BAP2-1-D	CAGTATAGCGACCAGCA
BAP2-2-U	TGCCCAGATGCGAAGT
BAP2-2-D	TGGTCCATTTGCCCAC
GDH1-1-U	AACGGTATCGACCCTAT
GDH1-1-D	AACCTCAGTGGCAAATC
GDH1-2-U	CAGACTAAACTGGCTGACG
GDH1-2-D	GCCTCCTTCGCAAACT
ADO80-1-U	CATTATCTTCGGCTCAAA
ADO80-1-D	CAAGACTGTCAAGGAGGG
ADO80-2-U	GCAGACCGATACCAGG
ADO80-2-D	CCCGTAACAAGGGAAA
CAN1-1-U	TGCCTTTGATAGTGCC
CAN1-1-D	CTAATACCTGGAATGCTG
CAN1-2-U	GGTATAAATGGGCTCG
CAN1-2-D	GTCGGTGGTCTCAACA
BAT2-1-U	TGTCGCCGCCGTCAATAA
BAT2-1-D	CTGAGCGAGACGAAATAC
BAT2-2-U	ATGCGTCAATCGTATGTG
BAT2-2-D	TTTCCTCTATGTCCTCCC

GAD1-1-U	AAGAGGAACCACCTACCC
GAD1-1-D	CCTGAGCGAGACGAAAT
GAD1-2-U	ATCGCGTATTTCGTCTC
GAD1-2-D	CCAGCACCATTTAGTCAT
**Oligos used for real time qPCR**	
FBC6-U	ACAAAGCAGGCTCACAAG
FBC6-D	AAGGAGTATCCGCAGGTC
GAT1-U	AGCACGAACAGAGTCCCG
GAT1-D	GGTTATGCAAACGCCAAG
AGP1-U	AATTGATCGGAATTGTATGG
AGP1-D	CAACATTAGTGACTGCTGCTT
BAP2-U	GAGGAGTATGCGTTGAAAT
BAP2-D	AGGTATTGGGACTGGTCTT
GDH1-U	AGACAAGTTCACGGAAGGA
GDH1-D	ACACCCAGAATACAGAAAGG
ADO80-U	TGACCACGACAACGAGAA
ADO80-D	CTTTGGGCGAATGAGAAT
CAN1-U	GGCGTTGGTCAGAGGTGT
CAN1-D	TGGAGGATGGCATAGGTG
BAT2-U	GCTGCGATTGTTTCTCCC
BAT2-D	TTGCCATGCTCAGTCTCG
GAD1-U	GTACCGCTGGTAGCCTTCA
GAD1-D	ATCCATCCGTTGCCTTTG
